# Progestin plus metformin improves outcomes in patients with endometrial hyperplasia and early endometrial cancer more than progestin alone: a meta-analysis

**DOI:** 10.3389/fendo.2023.1139858

**Published:** 2023-06-21

**Authors:** Fengping Shao, Yinguang Li, Yunhe Zhao

**Affiliations:** Department of Obstetrics and Gynecology, The First Affiliated Hospital, Sun Yat-sen University, Guangzhou, Guangdong, China

**Keywords:** metformin, progestin, hyperplasia, cancer, complete response, relapse, obstetrical outcomes

## Abstract

**Objective:**

Progestin based therapy is the preferred option for fertility-sparing treatment of reproductive-age women with preserved fertility in endometrial hyperplasia (EH) or early endometrial cancer (EEC). Our objective was to investigate whether metformin could enhance the efficacy of progestin-based therapies by meta-analysis.

**Methods:**

We conducted a meta-analysis of randomized or non-randomized controlled trials by searching of PubMed, Embase, Web of science, and Cochrane database from inception to November 8, 2022. The results of enrolled studies were pooled using meta-analysis to estimate the effect of progestin plus metformin on remission, recurrence, pregnancy rate and live birth rate.

**Results:**

In the analysis of progestin administered systemically or locally, complete response (CR) was significantly higher in progestin plus metformin versus progestin alone in the EH group (pooled OR 2.08, 95% CI 1.29 to 3.34, P=0.003), in the EEC group (pooled OR 1.86, 95% CI 1.13 to 3.05, P=0.01), but not in EEC and EH group (pooled OR 1.46, 95% CI 0.97 to 2.21, P=0.07). In the analysis of progestin administered systemically, complete response was improved in progestin plus metformin versus progestin alone, in the EH group (pooled OR 2.47, 95% CI 1.45 to 4.21, P=0.0009), in the EEC group (pooled OR 2.09, 95% CI 1.18 to 3.71, P=0.01), and in the EEC and EH group (pooled OR 2.03, 95% CI 1.16 to 3.54, P=0.01). The relapse rates of patients with EEC and EH were not different (pooled OR 0.54, 95% CI 0.24 to 1.20, P=0.13). For obstetric outcomes, the addition of metformin improved pregnancy rate (pooled OR 1.55, 95% CI 0.99 to 2.42, P=0.05), but not live birth rate (pooled OR 0.95, 95% CI 0.45 to 2.01, P=0.89).

**Conclusion:**

For fertility-sparing management, compared to progestin alone, the outcomes of patients with endometrial hyperplasia and early endometrial cancer were more improved with progestin plus metformin because progestin plus metformin increases the rate of remission and pregnancy.

## Introduction

In the United States, endometrial cancer is the most common gynecologic malignancy, with nearly 63,246 new cases diagnosed in 2022, and in China, its incidence is in the second place of gynecologic malignancies, with nearly 84,520 new cases diagnosed in 2022 (1). Endometrial hyperplasia (EH) is a common gynecological disease in reproductive-age women, defined as hyperplasia of the endometrial glands with irregular size and morphology, with or without atypical cells, whose main clinical manifestation is abnormal uterine bleeding, which can easily develop into endometrial cancer and seriously affect fertility.

Hormonal therapy, usually administered to promote remission and allow pregnancy, plays a dominant role in the fertility-sparing management. However, progestin therapy may not be the optimal regimen, with remission probabilities of 12 and 24 months were 78.0% and 81.4%, respectively, while the recurrence probabilities were up to 9.6% and 29.2%, respectively (2).

Metformin is a drug commonly used to treat patients with diabetes, but also plays a role in gynecologic endocrine disorders, such as improving the menstrual pattern, restoring ovulation, increasing pregnancy rates, lowering serum androgen levels, and reducing the risk for cardiovascular disease in women with polycystic ovary syndrome (3). Metformin therapy has been found to possibly help reverse atypical endometrial hyperplasia to normal endometrial histology, reduce the risk of endometrial abnormality (3, 4) and decrease biomarkers of cell proliferation associated with tumor progression, and improve overall survival in endometrial cancer (5).

As the age of women with reproductive requirements gradually increases, the occurrence of endometrial hyperplasia and endometrial cancer will severely impair the fertility of these older women. Hysterectomy in reproductive-age women can be greatly avoided only if conservative therapy is able to achieve satisfactory rates of disease remission and recurrence. Some previous meta-analyses have evaluated the efficacy of metformin in endometrial cancer or endometrial hyperplasia (6, 7). In 2017, a cochrane systematic review reported that there is insufficient evidence to support or refute the use of metformin alone or in combination with progestin (7); In 2021, a meta-analysis published by Jennifer Chae-Kim et al. showed that the addition of metformin to a progestin-based therapy reduced the rate of disease recurrence, but it failed to improve remission rates, pregnancy rates and live birth rates in patients (6). However, recent studies demonstrated that progestin plus metformin could improve both the rate of disease remission in endometrial hyperplasia (8) and in early endometrial cancer (9). Recently, with the publication of several studies (8–13), it is essential to further evaluate whether metformin is beneficial in improving the prognosis of endometrial cancer and endometrial hyperplasia. This meta-analysis is intended to integrate recent clinical studies to assess the benefits of metformin combination with progestin therapy in women suffering from early endometrial cancer or endometrial hyperplasia.

## Methods

### Search strategy

We conducted a meta-analysis to assess the efficacy of the combination of progestin and metformin in the treatment of endometrial hyperplasia and early endometrial cancer, in accordance with the recommendations of the PRISMA 2020 statement (14). We conducted a comprehensive systematic search of PubMed, Embase, Web of science, and Cochrane for all clinical studies including randomized controlled trials or non-randomized controlled trials, prospective studies or retrospective studies from inception to November 8, 2022 and up to May 17, 2023 in the final update. We searched the database using “metformin” and “hyperplasia or cancer” and “endometrial” as search terms in all fields. The study protocol was registered in PROSPERO with the registration number CRD42022373842.

### Selection criteria

The type of clinical study was not limited to prospective randomized controlled trials, however, to be eligible, the following conditions need to be met. Firstly, the study population: atypical or non-atypical endometrial hyperplasia, early endometrial cancer in a reproductive age group of women, who prefer to maintain fertility with endocrine therapy. Secondly, conservative treatment regimens involve the progestin alone, progestin in combination with metformin, and also, progestin types including megestrol (MA), medroxyprogesterone (MPA), norethisterone (NET), depo-medroxyprogesterone acetate (DMPA) and levonorgestrel-releasing intrauterine device (LNG-IUD), regardless of medication dose and duration of administration. Thirdly, the primary outcome: disease remission, and secondary outcomes: disease recurrence, clinical pregnancy rate, live birth rate and adverse reactions. Studies were excepted if they were literature reviews and meta-analyses, case reports, basic science papers and study protocol; also, if clinical studies did not cover progestin treatment alone and combination progestin with metformin for endometrial hyperplasia, or early endometrial cancer, they were excluded. The identical study, which may appear in multiple articles or different publications, was considered for the analysis of the one trial that presented the most complete data.

### Data extraction

Two investigators independently screened the title and abstract of each eligible paper, and reviewed the full text and even the supplementary information if necessary, and collected data using a pilot-tested data extraction sheet. If there were any disagreements, they were resolved through discussion and consultation. The following information was extracted from each selected trial and collected in extraction sheet: authors, year of publication, location of data source, prospective or retrospective, type of disease: endometrial hyperplasia with or without atypical, early stage endometrial cancer, number of patients in each treatment group (Prog-Met and Prog), number of patients in complete response, relapse, pregnancy and live birth, number of patients by EEC and EH, number of patients by BMI(body mass index), type of progestin, dose of metformin administered, follow-up time and adverse event.

### Quality assessment of the studies

Since two study types, randomized controlled trials and retrospective cohort studies, were enrolled, Cochrane Collaboration Risk of Bias Tool and Newcastle-Ottawa Scale were used to assess their quality separately. Cochrane Risk of Bias Tool consists of random sequence generation; allocation concealment; blinding of participants and personnel to the study protocol; blinding of outcome assessment; incomplete outcome data; and selective reporting (15), and Newcastle-Ottawa scale consists of three criteria: selection, comparability, and outcome assessment (16).

### Statistical analysis

The primary objective of the meta-analysis was complete response of patients in Prog-Met group and Prog group. The second objectives were relapse rates, clinical pregnancy rates, live birth rates and adverse events between two groups. Subgroup analysis was conducted by patient characteristics such as age, BMI, PCOS (polycystic ovary syndrome), and diabetes mellitus, if the data was available. P ≤ 0.10 or I2≥50% indicated significant heterogeneity by Cochran’s Q test and I^2^ statistics. If heterogeneity was not present, a fixed-effects model was used (P>0.10 and I2<50%) (17), otherwise, a random-effects model was used (P ≤ 0.10 or I2≥50%) (18). Data are shown as odds ratio (ORs) with 95% confidence intervals (CIs). The results were considered statistically significant if P value < 0.05. All analysis was carried out using performed using Review Manager 5.3 (Cochrane Collaboration, Copenhagen, Denmark) and the graphs were then optimized in R statistical computing software.

## Results

According to the search terms, the initial search resulted in 1680 reports, and by removing 765 duplicates, 902 publications that failed to meet the inclusion criteria, the final 13 trials were included (**Supplementary Figure 1**). Assessment of risk of bias for randomized controlled trials is presented in **Supplementary Figure 2**, and quality assessment of retrospective cohort studies is presented in **Supplementary Table 1**.

Among the 13 studies, 1 study was presented as a conference in 2020 (19), and finally as an article in 2023 (20), and 5 studies enrolled both patients with EH and patients with EEC (19–24), and 5 studies focused only on patients with EH (8, 11, 12, 25, 26), and 2 studies enrolled only patients with EEC (9, 10). Six studies were prospective trials (9, 11, 12, 23, 26, 27), while seven were retrospective trials (8, 10, 19–22, 24, 25). The study published by Matsuo et al. was a clinical trial with a predominantly obese population, with BMI >25 kg/m^2^ accounting for 92.6% of endometrial hyperplasia patients (25). There are two routes of progestin therapy, with systemic administration including oral medroxyprogesterone acetate, megestrol acetate, norethindrone, depo-medroxyprogesterone acetate, and local administration *via* a levonorgestrel-releasing intrauterine device. The route of progestin therapy were systemic administration in eight studies (8, 12, 19, 20, 22–24, 26), and local administration alone in two studies (11, 27), and systemic administration combined with local administration in one studies (10), and systemic or local administration in two studies (21, 25). The dose of metformin was administered in a range from 500 mg/d (21) to 2,500 mg/d (20, 25), with the commonly administered dose of 1,000 mg/d and 1,500 mg/d. Twelve studies provided follow-up times for the assessment of disease remission rates, ranging from 3 months (26) to 32.5 months (24). The characteristics of the 13 selected trials are summarized in **Table 1**.

**Table 1:** Characteristics of the eligible trials in the meta-analysis.

Author/year	Type of study	No. of Patients (Prog-Met/Prog)	Progestin type	Metformin dosage	Histological diagnosis	Reported outcomes	Time for assessing complete response	Time for assessing relapse
Shan 2014	Randomized trial	8/8	MA	1500 mg/d	AEH	Remission, relapse, pregnancy rate, live birth rate, adverse effect	12 weeks	12 months
Zhou 2015	Retrospective trial	9/23	MPA/MA	750 mg/d	AEH,EEC	Remission, relapse, pregnancy, live birth	32.5 months	34.0 weeks after CR
Mitsuhashi 2019	Retrospective trial	63/23	MPA	750-2250 mg/d	AEH,EEC	Remission, relapse, 5 year recurrence free survival, pregnancy rate, live birth rate, adverse effect	18 months	57 months
Acosta-Torres 2020	Retrospective trial	34/58	MPA/MA/LNG-IUD	500-1000 mg/d	AEH,EIH,EEC	Remission, relapse, live births, 5 year recurrence free survival	17.9 months	28.4 months
Matsuo 2020	Retrospective trial	51/194	MPA/MA/NET/DMPA /LNG-IUD	unavailable	AEH	Remission	12 months	NR
Yang 2020	Randomized trial	67/66	MA	1500 mg/d	AEH,EEC	Remission, relapse, pregnancy, live birth rate, adverse effect	32 weeks	33.4 months after CR
Tehraniana 2020	Randomized trial	29/27	MA	1000 mg/d	NAEH	Remission	3 months	NR
Janda 2021	Randomized trial	42/33	LNG-IUD	1000 mg/d	AEH,EEC	Remission	6 months	NR
Ravi 2021	Randomized trial	25/135	LNG-IUD	1000 mg/d	NAEH	Remission, adverse effect	6 months	NR
Pino 2022	Retrospective trial	24/20	LNG-IUD+MA	1500 mg/d	EEC	Remission, relapse, pregnancy rate, live birth rate	12 months	45 months
Kong 2022	Retrospective trial	81/138	MPA/MA	1500 mg/d	AEH	Remission, pregnancy rates, live birth rate, abortion rate, adverse effect	8/12 weeks	NR
Yuan 2022	Randomized trial	60/60	MPA	1500 mg/d	EEC	Remission, pregnancy rates, live birth rate, adverse effect	NR	NR
Tsuda 2020 Ushijima 2023	Retrospective trial	46/314	MPA	750-2500 mg/d	AEH,EEC	Remission, relapse, pregnancy, live birth rate, adverse effect	2290 days	2290 days

MPA, medroxyprogesterone acetate; MA, megestrol acetate; NET, norethindrone; DMPA, depo-medroxyprogesterone acetate. LNG-IUD, levonorgestrel-releasing intrauterine device; EAH, endometrial atypical hyperplasia; EC, endometrial cancer; EIH, endometrial intraepithelial neoplasia; EEC, early-stage endometrial cancer; NAEH, non-atypical endometrial hyperplasia; CR, complete response; Complex atypical hyperplasia (CAH) on pathology reports were categorized as AEH; NR, no report.

### Meta-analysis in patients with endometrial hyperplasia

**Figure 1:**
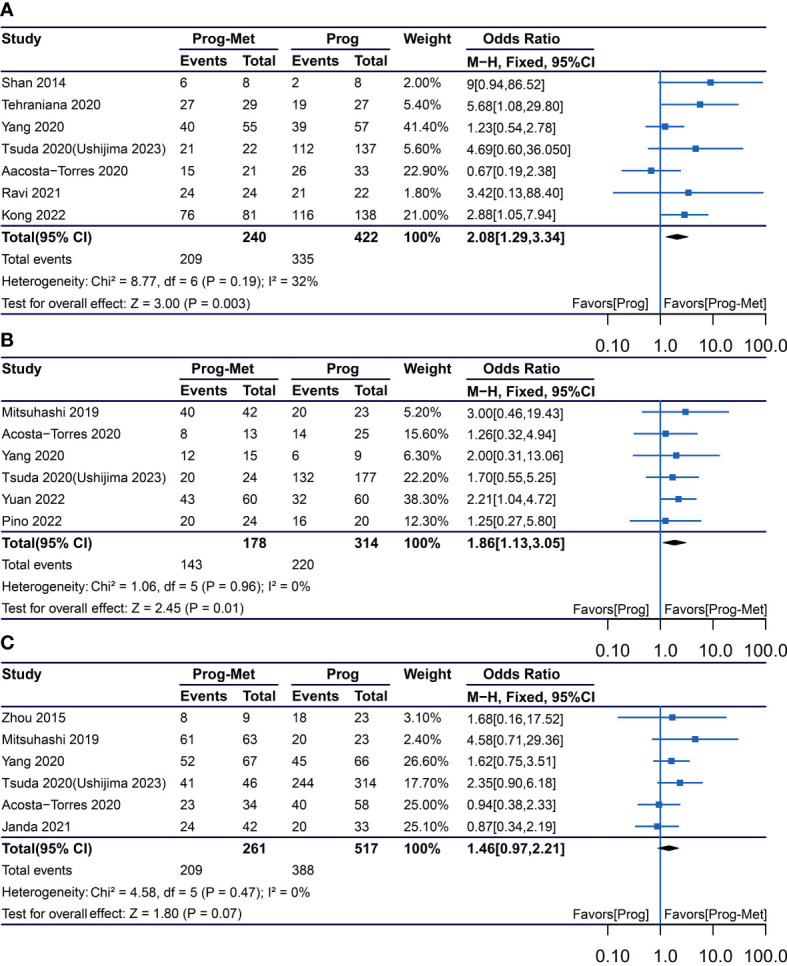
Complete response comparing Prog-Met versus Prog by administering progestin systemically and locally in subgroups. **(A)** Endometrial hyperplasia, **(B)** early-stage endometrial cancer, **(C)** endometrial hyperplasia and early-stage endometrial cancer. (Effect size is presented as odds ratio with 95% confidence interval. Odds ratio >1 means that progestin combined with metformin is superior to progestin. Prog, progestin; Met, metformin).

**Figure 2:**
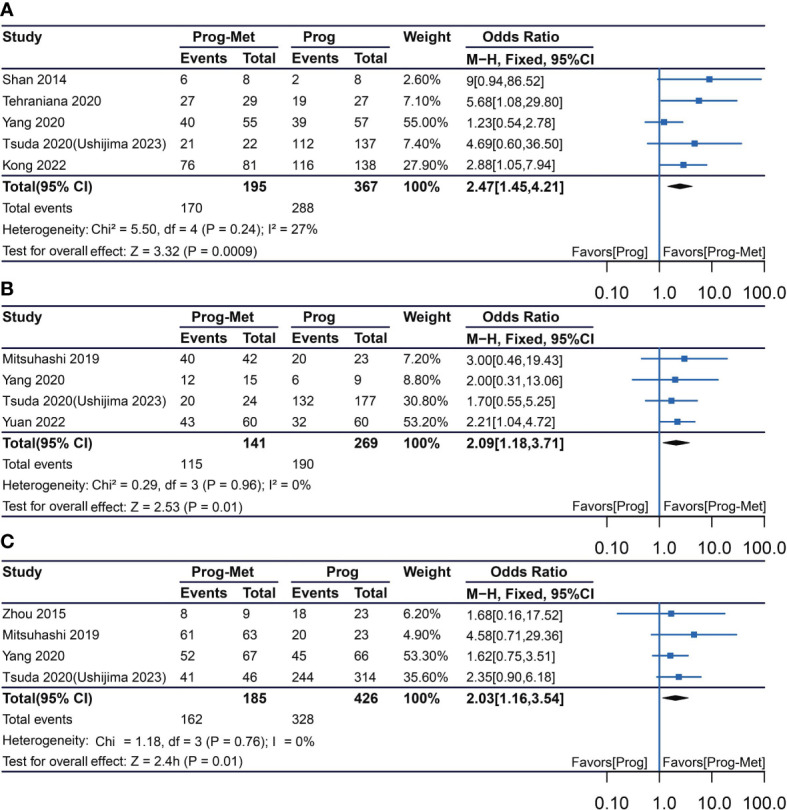
Complete response comparing Prog-Met versus Prog by administering progestin systemically in subgroups. **(A)** Endometrial hyperplasia, **(B)** early-stage endometrial cancer, **(C)** endometrial hyperplasia and early-stage endometrial cancer. (Effect size is presented as odds ratio with 95% confidence interval. Odds ratio >1 means that progestin combined with metformin is superior to progestin. Prog, progestin; Met, metformin).

In the mixed analysis (where progestin was administered systemically or locally) enrolling seven studies, complete remission of disease with progestin combined with metformin was statistically significantly better than with progestin alone (pooled OR 2.08, 95% CI 1.29 to 3.34, P=0.003), with 87.1% (209/240) and 79.4% (335/422) of women achieved complete remission in each group, respectively (**Figure 1A**). In the subgroup analysis (where progestin was administered systemically only) with five studies enrolling, the odds ratio was statistically elevated to 2.47(95% CI 1.45 to 4.21) with a P value of 0.0009 (**Figure 2A**). In subgroup of overweight population extracted from 3 studies, no difference was demonstrated between combination therapy and progestin alone (pooled OR 0.97, 95% CI 0.52 to 1.79, P=0.92) (**Supplementary Figure 3**).

### Meta-analysis in patients with early endometrial cancer

In the mixed analysis included five studies showed that complete disease remission was statistically better for the Prog-Met versus Prog (pooled OR 1.86, 95% CI 1.13 to 3.05, P=0.01), with 80.3% (143/178) and 70.1% (220/314) of women achieved complete remission in each group, respectively (**Figure 1B**). At subgroup analysis, the P value (P=0.001) for odds ratio (pooled OR 2.09) was also statistically significant, meaning that progestin plus metformin was superior to progestin alone. (**Figure 2B**).

### Meta-analysis of patient with early endometrial cancer and endometrial hyperplasia

**Figure 3:**
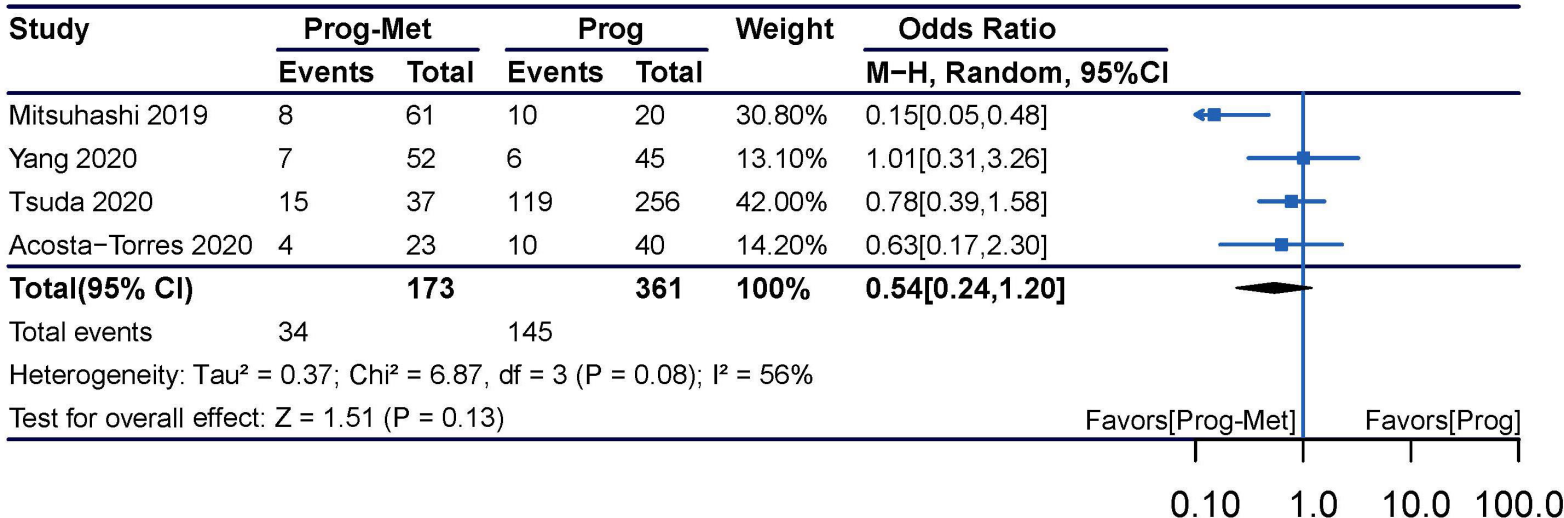
Relapse comparing Prog-Met versus Prog in endometrial hyperplasia and early-stage endometrial cancer. (Effect size is presented as odds ratio with 95% confidence interval. Odds ratio >1 means that progestin is superior to progestin combined with metformin. Prog, progestin; Met, metformin).

The results enrolling six studies for mixed analysis indicated that complete disease remission were similar between Prog-Met group and Prog group (pooled OR 1.46, 95% CI 0.97 to 2.21, P=0.07), with 80.1% (209/261) and 75.0% (388/517) of women achieved complete remission in each group, respectively (**Figure 1C**). After removing one study with local administration *via* LNG-IUD (21), five studies remained for subgroup analysis resulting to show that the odds ratio was 2.03(95% CI 1.16 to 3.54), when comparing the two groups with a p-value of 0.01 meaning statistically significant (**Figure 2C**). The Prog-Met group was not able to reduce the risk of relapse, when compared to the Prog group by pooled four studies with using random effect model (pooled OR 0.54, 95% CI 0.24 to 1.20, P=0.13), with 19.7% (34/173) and 40.2% (145/361) of women relapsed after achieving complete remission in each group, respectively (**Figure 3**). When choosing a fixed effects model, the results showed that the Prog-Met group was able to reduce the risk of relapse, when compared to the Prog group (pooled OR 0.60, 95% CI 0.37 to 0.97, P=0.04) (Figure not provided).

### Clinical pregnancy rate and live birth rate

**Figure 4:**
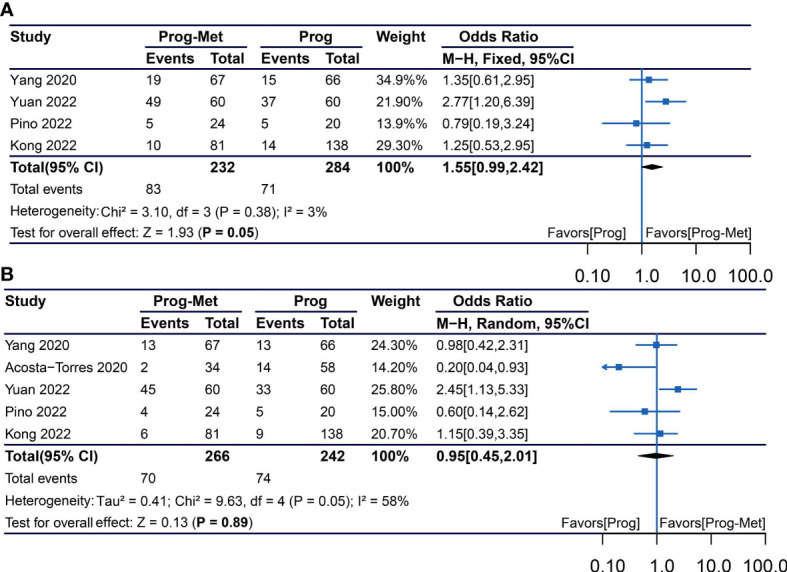
Obstetric outcomes comparing Prog-Met versus Prog for subgroups. **(A)** Clinical pregnancy rate and live birth rate **(B)**. (Effect size is presented as odds ratio with 95% confidence interval. Odds ratio >1 means that progestin combined with metformin is superior to progestin. Prog, progestin; Met, metformin).

Using the total number of participants in each treatment group as the denominator, our meta-analysis showed that compared with progestin alone, the addition of metformin in patients with endometrial hyperplasia and early endometrial cancer may improve clinical pregnancy rate (pooled OR 1.55, 95% CI 0.99 to 2.42, P=0.05), but not increase live birth rate (pooled OR 0.95, 95% CI 0.45 to 2.01, P=0.89) (**Figure 4**).

### Evaluation of adverse reactions

Few studies had specifically reported adverse reactions. According to 3 studies published by Yang et al, Yuan et al. and Ravi, R. D. et al, the adverse reactions were not severe in both Prog-Met group and Prog group, and the common adverse reactions of metformin were gastrointestinal reactions. The addition of metformin significantly increased the occurrence of gastrointestinal reactions (pooled OR 2.91, 95% CI 1.57 to 5.40, P=0.008) and nausea (pooled OR 3.06, 95% CI 1.20 to 7.78, P=0.02), but not abdominal pain or insomnia (**Supplementary Figure 4**).

## Discussion

Considerable basic and clinical research has supported that metformin plays an important role in fertility-preserving treatment. Metformin sensitizes endometrial cancer cells even progestin-resistant EC cells to progestin by promoting progesterone receptor (28), downregulating glyoxalase I expression (29), downregulating glyoxalase I expression (30). Also, metformin alleviates endometrial hyperplasia by downregulating the expression levels of urothelial cancer associated 1, transforming growth factor−β and protein kinase B (31). Progestin-assisted metformin treatment was associated with a lower rate of disease recurrence for atypical endometrial hyperplasia and endometrial cancer (6, 32).

We expanded the newly published study to perform an integration and stratification analysis. Our results showed that combination therapy improved remission rate and pregnancy rate in patients with endometrial hyperplasia and early stage endometrial cancer, but not the relapse rate. Interestingly, the study published by Raffone A et al. (33) suggested that mismatch repair proteins appears to be able to influence disease recurrence. Noteworthy, when progestin was administered systemically, the difference in disease remission rates became more significant between the two groups. In our study, there was a significant difference between the results and those published by Jennifer Chae-Kim et al. (6), which was attributed to the inclusion of more studies in this meta-analysis.

Progestin-based therapy is the conservative treatment strategy to preserve fertility in patients with early stage endometrial cancer and atypical endometrial hyperplasia, which includes both systemic administration of oral medroxyprogesterone acetate, megestrol acetate, norethindrone, depo-medroxyprogesterone acetate, and local administration of LNG-IUD. However, remission rates of women with early endometrial cancer and atypical complex hyperplasia managed with progestin or LNG-IUD alone were just 71% and 76%, respectively (34), which were not so ideal that it requires improvement. Our study observed remission rates of 87.1% and 80.3% in patients with EH and EEC treated with metformin combined with progestin, respectively, both of which were higher than progestin alone with 79.4% and 70.1%, respectively. The study published by Casadio P et al. (35) showed that hysteroscopy combined with progestin resulted in more perfect disease remission rates and pregnancy outcomes, for which multiple hysteroscopies and multiple endometrial biopsies were required, which may be a difficulty for some patients. Anyway, these information would be particularly informative in helping doctors make clinical decisions for their patients.

It is well known that patients with obesity and polycystic ovary syndrome are highly represented among patients with endometrial hyperplasia and endometrial cancer. With several studies analyzed the relationship between obesity status and disease response, our results by enrolling 3 studies (8, 22, 25) for meta-analysis suggested that there was no improvement in disease remission rate for overweight population treated with combination systemic progestin and metformin. However, two studies (8, 22) have claimed that metformin combined with systemic progestin has therapeutic value in patients with a BMI ≥25 kg/m^2^, because of the effect of metformin in reducing insulin resistance (8), also resulting in weight loss in patients to counteract the metabolic effects of systemic progestins (22). Noteworthy, one of these three studies (25) suggested that by subgroup analysis concurrent metformin may possibly offer treatment benefit, when used with the levonorgestrel-releasing intrauterine device rather than systemic administration, because the former keeping the anti-inflammatory effects of metformin for overweight patients, although the overall data do not confirm the benefit of metformin. Systemic progestin may contribute to the elevation of inflammatory cytokine by increasing body weight, which in turn counteracts the efficacy of metformin (25). Consequently, it remains inconclusive whether metformin has therapeutic value and whether it depends on the progestin route in obese patient.

For patients with PCOS, two studies have shown that PCOS status is not a risk factor for disease prognosis (10, 21), and another study found that PCOS patients had a significantly higher CR rate in the Prog-Met group compared to the Prog group (p=0.028) (8). Metformin may reverse endometrial dysfunction in PCOS women, and improve pregnancy outcomes in obese and/or insulin resistant patients (4). Therefore, there is a demand for prospective randomized controlled trials with large numbers of participants, and even more so, clinical trials stratified by patient characteristics. There is an ongoing randomized controlled trial to investigate metformin in combination with MPA for fertility-sparing treatment, in which the primary objective is 3-year relapse-free survival (RFS) and secondary objectives are response rate to MPA therapy, pregnancy rate and live birth rate, toxicity evaluation and changes in insulin resistance and body mass index (FELICIA trial) (36).

For pregnancy outcomes in most studies, detailed data were unavailable, and there was variation in the denominators used to calculate pregnancy and live birth rates across studies, in which the number of total participants, the number of patients with remission of disease, or the number of patients trying conceive. Distinct from previous studies (6), in our meta-analysis, we pooled the total number of participants in each treatment group as the denominator and showed that the pregnancy rate may be higher in the Prog-Met group compared to the Prog group.

Adverse reactions are an important consideration in the long-term delivery of combination therapy strategies. Due to insufficient data on reported adverse events, our study only analyzed adverse reactions such as gastrointestinal reactions, nausea, insomnia, and abdominal pain. The results showed that Prog-Met treatment increased gastrointestinal adverse reactions and nausea, but the extent of these symptoms was mild.

The analysis has some limitations, mainly as follows: Firstly, the selected studies were mostly retrospective, with small samples involved. Secondly, these studies were non-consistent in their observational purpose, and several were unavailable to adequately cover disease remission rates, recurrence rates, pregnancy outcomes, and adverse effects, and for disease remission may lack consistent pathological assessment. Thirdly, there was inconsistency in the histological diagnosis of patients in these studies, with both endometrial hyperplasia and endometrial cancer, and in addition endometrial hyperplasia included two histological types, atypical cell and without atypical cell. Fourthly, the differences are manifested in patient characteristics, but also in the diversity of progestin agent and also in the diversity of metformin dosage. For example, in this study by Acosta-Torres et al, women in the Prog-Met group were more often characterized by a younger, higher BMI, DM (diabetes mellitus) and PCOS (21); in another study by Zhou et al, only patients with elevated HBA1C (glycosylated hemoglobin A1C) were treated with metformin (24). Finally, it was unavailable to conduct subgroup analyses based on patients’ characteristics, such as age, body mass index, reproductive status, and comorbid conditions.

## Conclusion

Our meta-analysis found that the addition of metformin to progestin-based therapy contributed to the improvement of disease remission rate in women with endometrial hyperplasia and early stage endometrial cancer. Metformin failed to improve disease recurrence rate. When progestin was administered systemically, the difference in disease remission rates between the Prog-Pet and Prog groups became more pronounced. Pregnancy rates may be higher in the Prog-Met group, but live birth rates were similar in both groups.

## Data availability statement

The original contributions presented in the study are included in the article/**Supplementary Material**. Further inquiries can be directed to the corresponding author.

## Author contributions

FS and YL: Conceptualization, Methodology, Software, Validation, Data curation; FS: Writing-Original draft preparation, Diagram processing; FS and YZ: Supervision, Writing- Reviewing and Editing. All authors contributed to the article and approved the submitted version.

## References

[1] Xia C Dong X Li H Cao M Sun D He S. Cancer statistics in China and united states, 2022: profiles, trends, and determinants. Chin Med J (Engl) (2022) 135(5):584–90. doi: 10.1097/cm9.0000000000002108 PMC8920425 35143424

[2] Koskas M Uzan J Luton D Rouzier R Darai E. Prognostic factors of oncologic and reproductive outcomes in fertility-sparing management of endometrial atypical hyperplasia and adenocarcinoma: systematic review and meta-analysis. Fertil Steril (2014) 101(3):785–+. doi: 10.1016/j.fertnstert.2013.11.028 24388202

[3] Palomba S Falbo A Zullo F Orio F Jr. Evidence-based and potential benefits of metformin in the polycystic ovary syndrome: a comprehensive review. Endocr Rev (2009) 30(1):1–50. doi: 10.1210/er.2008-0030 19056992

[4] Palomba S Piltonen TT Giudice LC. Endometrial function in women with polycystic ovary syndrome: a comprehensive review. Hum Reprod Update (2021) 27(3):584–618. doi: 10.1093/humupd/dmaa051 33302299

[5] Meireles CG Pereira SA Valadares LP Rêgo DF Simeoni LA Guerra ENS. Effects of metformin on endometrial cancer: systematic review and meta-analysis. Gynecologic Oncol (2017) 147(1):167–80. doi: 10.1016/j.ygyno.2017.07.120 28760367

[6] Chae-Kim J Garg G Gavrilova-Jordan L Blake LE Kim TT Wu Q. Outcomes of women treated with progestin and metformin for atypical endometrial hyperplasia and early endometrial cancer: a systematic review and meta-analysis. Int J gynecological Cancer Off J Int Gynecological Cancer Soc (2021) 31(12):1499–505. doi: 10.1136/ijgc-2021-002699 34785524

[7] Clement NS Oliver TR Shiwani H Sanner JR Mulvaney CA Atiomo W. Metformin for endometrial hyperplasia. Cochrane Database Syst Rev (2017) 10(10):Cd012214. doi: 10.1002/14651858.CD012214.pub2 29077194 PMC6485333

[8] Kong WY Liu ZA Zhang N Wu X Zhao XB Yan L. A prospective cohort study of metformin as an adjuvant therapy for infertile women with endometrial complex Hyperplasia/Complex atypical hyperplasia and their subsequent assisted reproductive technology outcomes. Front Endocrinol (Lausanne) (2022) 13:849794. doi: 10.3389/fendo.2022.849794 35846327 PMC9280669

[9] Yuan F Hu Y Han X Li Q. Metformin in combination with progesterone improves the pregnancy rate for patients with early endometrial cancer. Contrast Media Mol Imaging (2022) 2022:1961016. doi: 10.1155/2022/1961016 35854762 PMC9279044

[10] Pino I Iacobone AD Urbinati AMV Di Giminiani M Radice D Guerrieri ME. Fertility-sparing treatment for endometrial cancer: oncological and obstetric outcomes in combined therapies with levonorgestrel intrauterine device. Cancers (2022) 14(9):10. doi: 10.3390/cancers14092170 PMC9101107 35565299

[11] Ravi RD Kalra J Srinivasan R Bagga R Jain V Suri V. A randomized clinical trial of levonorgestrel intrauterine system with or without metformin for treatment of endometrial hyperplasia without atypia in Indian women. Asian Pac J Cancer Prev (2021) 22(3):983–9. doi: 10.31557/apjcp.2021.22.3.983 PMC8286694 33773565

[12] Tehranian A Ghahghaei-Nezamabadi A Arab M Khalagi K Aghajani R Sadeghi S. The impact of adjunctive metformin to progesterone for the treatment of non-atypical endometrial hyperplasia in a randomized fashion, a placebo-controlled, double blind clinical trial. J Gynecol Obstet Hum Reprod (2021) 50(6):101863. doi: 10.1016/j.jogoh.2020.101863 32652300

[13] Janda M Robledo KP Gebski V Armes JE Alizart M Cummings M. Complete pathological response following levonorgestrel intrauterine device in clinically stage 1 endometrial adenocarcinoma: results of a randomized clinical trial. Gynecologic Oncol (2021) 161(1):143–51. doi: 10.1016/j.ygyno.2021.01.029 33762086

[14] Page MJ McKenzie JE Bossuyt PM Boutron I Hoffmann TC Mulrow CD. The prisma 2020 statement: an updated guideline for reporting systematic reviews. Bmj (2021) 372:n71. doi: 10.1136/bmj.n71 33782057 PMC8005924

[15] Higgins JP Altman DG Gøtzsche PC Jüni P Moher D Oxman AD. The cochrane collaboration's tool for assessing risk of bias in randomised trials. Bmj (2011) 343:d5928. doi: 10.1136/bmj.d5928 22008217 PMC3196245

[16] Wells BS GA O'Connell D Peterson J Welch V Losos M Tugwell P. The Newcastle-Ottawa scale (Nos) for assessing the quality of nonrandomised studies in meta-analyses. Available at: https://www.ohri.ca/programs/clinical_epidemiology/oxford.asp.

[17] Mantel N Haenszel W. Statistical AspeXcts of the analysis of data from retrospective studies of disease. J Natl Cancer Inst (1959) 22(4):719–48. doi: 10.1093/jnci/22.4.719 13655060

[18] DerSimonian R Laird N. Meta-analysis in clinical trials. Control Clin Trials (1986) 7(3):177–88. doi: 10.1016/0197-2456(86)90046-2 3802833

[19] Tsuda N Ushijima K Mikami M Yamagami W Mitsuhashi A Shozu M. Trends and characteristics of fertility-sparing treatment for atypical endometrial hyperplasia and endometrial cancer in Japan: a survey by the gynecologic oncology committee of Japan society of obstetrics and gynecology. Gynecologic Oncol (2020) 159:336–7. doi: 10.1016/j.ygyno.2020.05.620 PMC10157339 36659833

[20] Ushijima K Tsuda N Yamagami W Mitsuhashi A Mikami M Yaegashi N. Trends and characteristics of fertility-sparing treatment for atypical endometrial hyperplasia and endometrial cancer in Japan: a survey by the gynecologic oncology committee of the Japan society of obstetrics and gynecology. J Gynecol Oncol (2023) 34(3):e38. doi: 10.3802/jgo.2023.34.e38 36659833 PMC10157339

[21] Acosta-Torres S Murdock T Matsuno R Beavis AL Stone RL Wethington SL. The addition of metformin to progestin therapy in the fertility-sparing treatment of women with atypical Hyperplasia/Endometrial intraepithelial neoplasia or endometrial cancer: little impact on response and low live-birth rates. Gynecol Oncol (2020) 157(2):348–56. doi: 10.1016/j.ygyno.2020.02.008 32085863

[22] Mitsuhashi A Habu Y Kobayashi T Kawarai Y Ishikawa H Usui H. Long-term outcomes of progestin plus metformin as a fertility-sparing treatment for atypical endometrial hyperplasia and endometrial cancer patients. J Gynecol Oncol (2019) 30(6):e90. doi: 10.3802/jgo.2019.30.e90 31576686 PMC6779615

[23] Yang BY Gulinazi Y Du Y Ning CC Cheng YL Shan WW. Metformin plus megestrol acetate compared with megestrol acetate alone as fertility-sparing treatment in patients with atypical endometrial hyperplasia and well-differentiated endometrial cancer: a randomised controlled trial. BJOG (2020) 127(7):848–57. doi: 10.1111/1471-0528.16108 31961463

[24] Zhou R Yang Y Lu Q Wang J Miao Y Wang S. Prognostic factors of oncological and reproductive outcomes in fertility-sparing treatment of complex atypical hyperplasia and low-grade endometrial cancer using oral progestin in Chinese patients. Gynecol Oncol (2015) 139(3):424–8. doi: 10.1016/j.ygyno.2015.09.078 26428941

[25] Matsuo K Mandelbaum RS Ciccone M Khoshchehreh M Pursuwani H Morocco EB. Route-specific association of progestin therapy and concurrent metformin use in obese women with complex atypical hyperplasia. Int J Gynecol Cancer (2020) 30(9):1331–9. doi: 10.1136/ijgc-2020-001362 PMC7521080 32376736

[26] Shan W Wang C Zhang Z Gu C Ning C Luo X. Conservative therapy with metformin plus megestrol acetate for endometrial atypical hyperplasia. J Gynecol Oncol (2014) 25(3):214–20. doi: 10.3802/jgo.2014.25.3.214 PMC4102740 25045434

[27] Janda M Robledo K Gebski V Armes J Alizart M Cummings M. Complete pathological response following levonorgestrel intrauterine device in clinically stage I endometrial adenocarcinoma: results of a randomized clinical trial. Gynecologic Oncol (2021) 162:S43–. doi: 10.1016/S0090-8258(21)00726-5 33762086

[28] Xie Y Wang YL Yu L Hu Q Ji L Zhang Y. Metformin promotes progesterone receptor expression via inhibition of mammalian target of rapamycin (Mtor) in endometrial cancer cells. J Steroid Biochem Mol Biol (2011) 126(3-5):113–20. doi: 10.1016/j.jsbmb.2010.12.006 21168492

[29] Jiang Y Chen X Wei Y Feng Y Zheng W Zhang Z. Metformin sensitizes endometrial cancer cells to progestin by targeting Tet1 to downregulate glyoxalase I expression. BioMed Pharmacother (2019) 113:108712. doi: 10.1016/j.biopha.2019.108712 30849641

[30] Zhang Z Dong L Sui L Yang Y Liu X Yu Y. Metformin reverses progestin resistance in endometrial cancer cells by downregulating gloi expression. Int J Gynecol Cancer (2011) 21(2):213–21. doi: 10.1097/IGC.0b013e318207dac7 21270604

[31] Guo M Zhou JJ Huang W. Metformin alleviates endometrial hyperplasia through the Uca1/Mir−144/Tgf−β1/Akt signaling pathway. Int J Mol Med (2020) 45(2):623–33. doi: 10.3892/ijmm.2019.4438 31894313

[32] Mitsuhashi A Sato Y Kiyokawa T Koshizaka M Hanaoka H Shozu M. Phase ii study of medroxyprogesterone acetate plus metformin as a fertility-sparing treatment for atypical endometrial hyperplasia and endometrial cancer. Ann Oncol (2016) 27(2):262–6. doi: 10.1093/annonc/mdv539 26578736

[33] Raffone A Catena U Travaglino A Masciullo V Spadola S Della Corte L. Mismatch repair-deficiency specifically predicts recurrence of atypical endometrial hyperplasia and early endometrial carcinoma after conservative treatment: a multi-center study. Gynecol Oncol (2021) 161(3):795–801. doi: 10.1016/j.ygyno.2021.03.029 33812697

[34] Wei J Zhang W Feng L Gao W. Comparison of fertility-sparing treatments in patients with early endometrial cancer and atypical complex hyperplasia: a meta-analysis and systematic review. Med (Baltimore) (2017) 96(37):e8034. doi: 10.1097/md.0000000000008034 PMC5604661 28906392

[35] Casadio P La Rosa M Alletto A Magnarelli G Arena A Fontana E. Fertility sparing treatment of endometrial cancer with and without initial infiltration of myometrium: a single center experience. Cancers (2020) 12(12):3571. doi: 10.3390/cancers12123571 33260382 PMC7760930

[36] Mitsuhashi A Kawasaki Y Hori M Fujiwara T Hanaoka H Shozu M. Medroxyprogesterone acetate plus metformin for fertility-sparing treatment of atypical endometrial hyperplasia and endometrial carcinoma: trial protocol for a prospective, randomised, open, blinded-endpoint design, dose-response trial (Felicia trial). BMJ Open (2020) 10(2):e035416. doi: 10.1136/bmjopen-2019-035416 PMC7050341 32114477

