# Supplementary figures and images for: Progestin plus metformin improves outcomes in patients with endometrial hyperplasia and early endometrial cancer more than progestin alone: a meta-analysis
Source: Front Endocrinol (Lausanne). 2023 Jun 21;14:1139858. doi: 10.3389/fendo.2023.1139858 (PMC10320576; doi:10.3389/fendo.2023.1139858)

## Supplementary Figure 1 Flow diagram for selection of studies

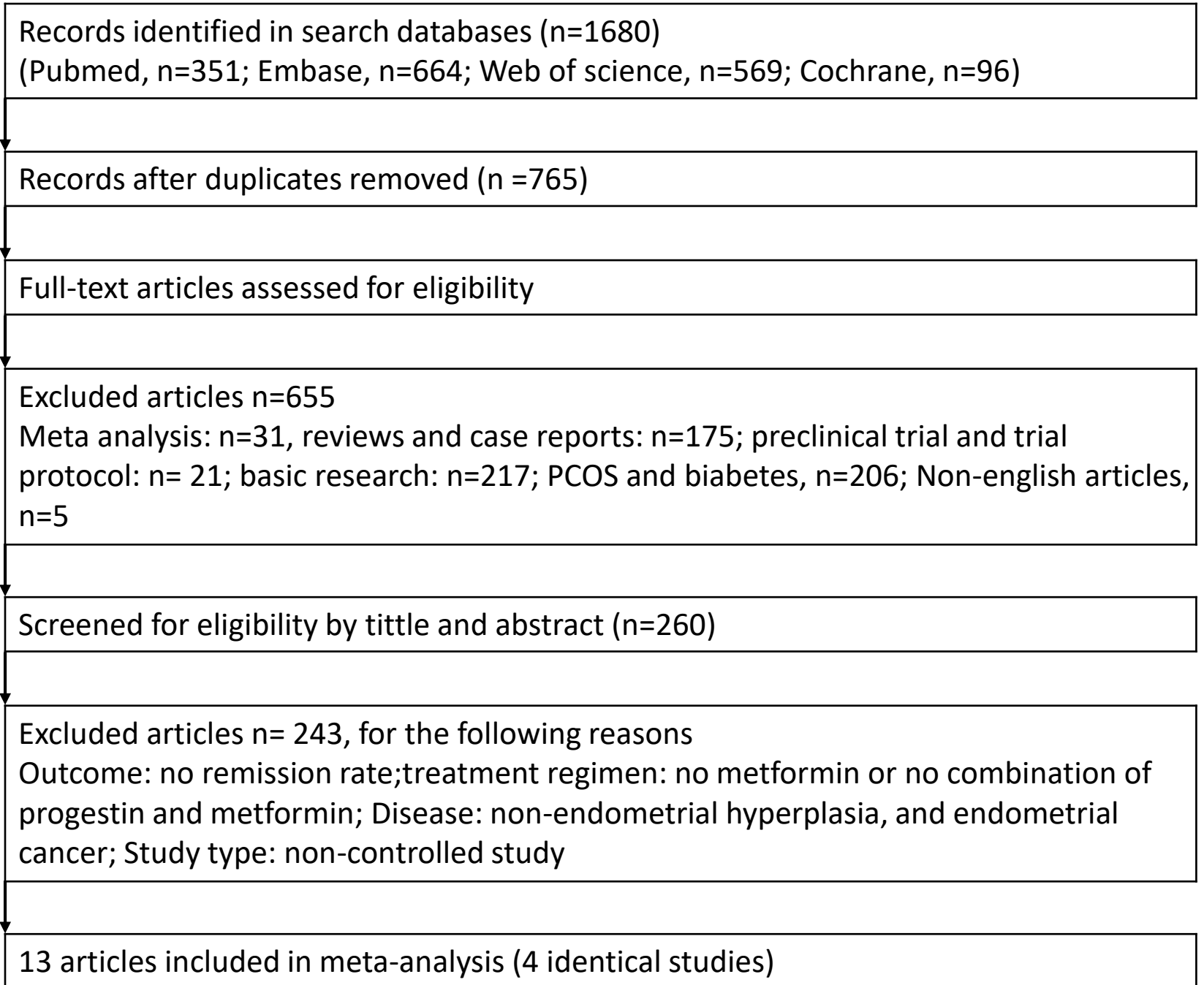

Supplement: Supplementary file 1 [file Image_1.pdf]

Supplementary Figure 2 Quality of randomized controlled trials by Cochrane Risk of Bias Tool

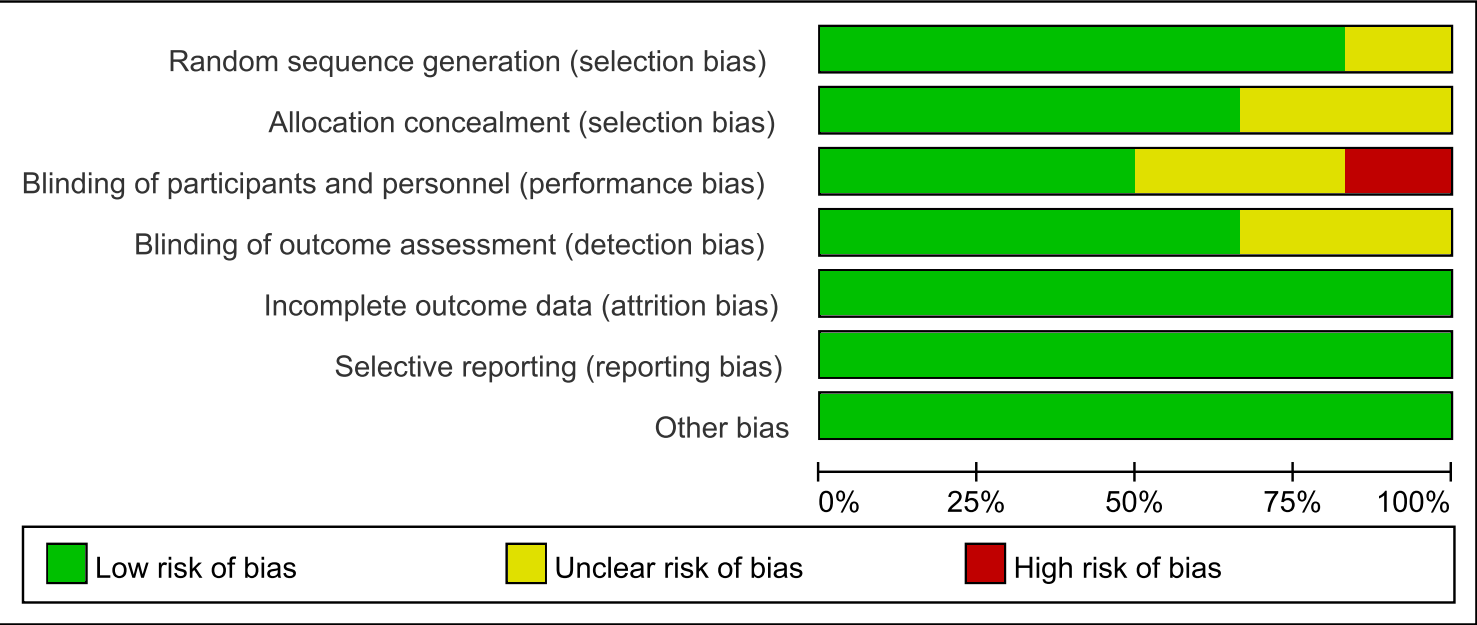

Supplement: Supplementary file 2 [file Image_2.pdf]
