# Supplementary material for: Progestin plus metformin improves outcomes in patients with endometrial hyperplasia and early endometrial cancer more than progestin alone: a meta-analysis
Source: Front Endocrinol (Lausanne). 2023 Jun 21;14:1139858. doi: 10.3389/fendo.2023.1139858 (PMC10320576; doi:10.3389/fendo.2023.1139858)

Supplementary Figure 3 Complete response comparing Prog-Met versus Prog in endometrial hyperplasia among overweight population. (Effect size is presented as odds ratio with 95% confidence interval. Odds ratio >1 means that progestin combined with metformin is superior to progestin. Prog, progestin; Met, metformin).

Study	Prog-Met		Prog		Weight	Odds Ratio
	Events	Total	Events	Total		M−H, Fixed, 95%CI
Matsuo 2020	38	51	156	194	80.40%	0.71[0.35,1.47]
Yang 2020	2	20	1	14	5.10%	1.44[0.12,17.67]
Kong 2022	51	54	61	69	14.50%	2.23[0.56,8.84]
**Total(95% CI)**		**125**		**277**	**100%**	**0.97[0.52,1.79]**
Total events	91		218			

Heterogeneity: Chi² = 2.20, df = 2 (P = 0.33); $I^2 = 9\%$

Test for overall effect: Z = 0.10 (**P = 0.92**)

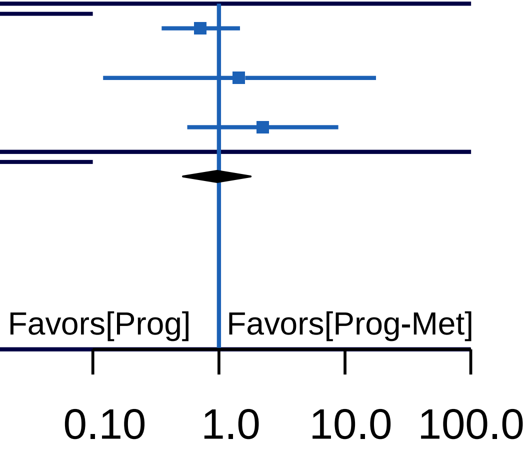

Supplement: Supplementary file 3 [file Image_3.pdf]
