# Supplementary material for: Progestin plus metformin improves outcomes in patients with endometrial hyperplasia and early endometrial cancer more than progestin alone: a meta-analysis
Source: Front Endocrinol (Lausanne). 2023 Jun 21;14:1139858. doi: 10.3389/fendo.2023.1139858 (PMC10320576; doi:10.3389/fendo.2023.1139858)

Supplementary Figure 4 Adverse reactions comparing Prog-Met versus Prog concerning gastrointestinal reactions, nausea, abdominal pain and insomnia. (Effect size is presented as odds ratio with 95% confidence interval. Odds ratio >1 means that adverse reactions are frequent in progestin combined with metformin compared to progestin. (Prog, progestin; Met, metformin)

**Study**	**Prog-Met**		**Prog**		**Weight**	**Odds Ratio**
	**Events**	**Total**	**Events**	**Total**		**M−H, Fixed, 95%CI**
Gastrointestinal reactions						
Yang 2020	23	76	9	74	51.10%	3,13[1.34,7.35]
Ravi 2021	10	25	3	26	14.20%	5.11[1.21,21.68]
Yuan 2022	8	60	5	60	34.80%	1.69[0.52,5.51]
**Total(95% CI)**	41	**161**	17	**160**	**100%**	**2.91[1.57,5.40]**
Total events						
Test for overall effect: Z = 3.39 (**P = 0.0007**)						
Nausea						
Yang 2020	8	76	4	74	67.30%	2.06[0.59,7.16]
Ravi 2021	10	25	3	26	32.70%	5.11[1.21,21.68]
**Total(95% CI)**	18	**101**	7	**100**	**100%**	**3.06[1.20,7.78]**
Total events						
Test for overall effect: Z = 2.34 (**P = 0.02**)						
Abdominal pain						
Yang 2020	14	76	12	74	67.80%	1.17[0.50,2.72]
Ravi 2021	5	25	6	26	32.20%	0.83[0.22,3.18]
**Total(95% CI)**	19	**101**	18	**100**	**100%**	**1.06[0.52,2.16]**
Total events						
Test for overall effect: Z = 0.16 (**P = 0.87**)						
Insomnia						
Yang 2020	2	76	3	74	39.20%	0.64[0.10,3.94]
Yuan 2022	5	60	5	60	60.80%	1.00[0.27,3.65]
**Total(95% CI)**	7	**136**	8	**124**	**100%**	**0.86[0.30,2.45]**
Total events						
Test for overall effect: Z = 0.28 (**P = 0.78**)						

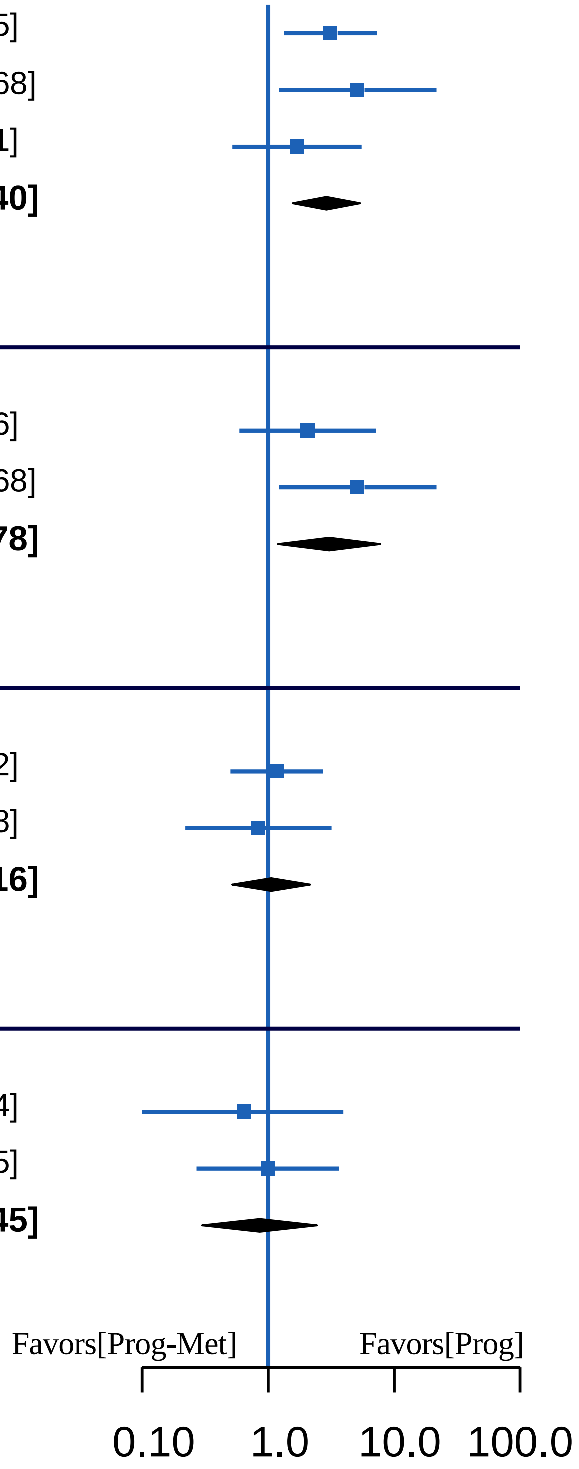

Supplement: Supplementary file 4 [file Image_4.pdf]
