# Supplementary material for: Progestin plus metformin improves outcomes in patients with endometrial hyperplasia and early endometrial cancer more than progestin alone: a meta-analysis
Source: Front Endocrinol (Lausanne). 2023 Jun 21;14:1139858. doi: 10.3389/fendo.2023.1139858 (PMC10320576; doi:10.3389/fendo.2023.1139858)
Supplement: Supplementary file 5 [file Table_1.docx]

| Study | Selection | Comparability | Outcome | Quality scores |
| --- | --- | --- | --- | --- |
| Rong Zhou 2015 | ☆☆☆ | ☆ | ☆☆☆ | 7 |
| Mitsuhashi 2019 | ☆☆☆ | ☆☆ | ☆☆☆ | 8 |
| Acosta-Torres 2020 | ☆☆☆☆ | ☆ | ☆☆☆ | 8 |
| Matsuo 2020 | ☆☆☆☆ | ☆ | ☆☆☆ | 8 |
| Tsuda 2020 (Ushijima 2023) | ☆☆☆ | ☆☆ | ☆☆☆ | 8 |
| Ida Pino 2022 | ☆☆☆☆ | ☆ | ☆☆☆ | 8 |
| Weiya Kong 2022 | ☆☆☆☆ | ☆☆ | ☆☆☆ | 9 |

Supplementary Table 1 Quality of retrospective cohort studies by Newcastle-Ottawa Scale
